# Oxidative stress alters mitochondrial bioenergetics and modifies pancreatic cell death independently of cyclophilin D, resulting in an apoptosis-to-necrosis shift

**DOI:** 10.1074/jbc.RA118.003200

**Published:** 2018-04-06

**Authors:** Jane A. Armstrong, Nicole J. Cash, Yulin Ouyang, Jack C. Morton, Michael Chvanov, Diane Latawiec, Muhammad Awais, Alexei V. Tepikin, Robert Sutton, David N. Criddle

**Affiliations:** From the Departments of § Cellular & Molecular Physiology and; ‡ Molecular and Clinical Cancer Medicine, Institute of Translational Medicine, University of Liverpool, Liverpool L69 3BX, United Kingdom

**Keywords:** pancreas, oxidative stress, mitochondrial permeability transition (MPT), cyclophilin D, bioenergetics, reactive oxygen species (ROS), apoptosis, necrosis (necrotic death), antioxidant, Acute Pancreatitis, Seahorse

## Abstract

Mitochondrial dysfunction lies at the core of acute pancreatitis (AP). Diverse AP stimuli induce Ca^2+^-dependent formation of the mitochondrial permeability transition pore (MPTP), a solute channel modulated by cyclophilin D (CypD), the formation of which causes ATP depletion and necrosis. Oxidative stress reportedly triggers MPTP formation and is elevated in clinical AP, but how reactive oxygen species influence cell death is unclear. Here, we assessed potential MPTP involvement in oxidant-induced effects on pancreatic acinar cell bioenergetics and fate. H_2_O_2_ application promoted acinar cell apoptosis at low concentrations (1–10 μm), whereas higher levels (0.5–1 mm) elicited rapid necrosis. H_2_O_2_ also decreased the mitochondrial NADH/FAD^+^ redox ratio and ΔΨ_m_ in a concentration-dependent manner (10 μm to 1 mm H_2_O_2_), with maximal effects at 500 μm H_2_O_2_. H_2_O_2_ decreased the basal O_2_ consumption rate of acinar cells, with no alteration of ATP turnover at <50 μm H_2_O_2_. However, higher H_2_O_2_ levels (≥50 μm) diminished spare respiratory capacity and ATP turnover, and bioenergetic collapse, ATP depletion, and cell death ensued. Menadione exerted detrimental bioenergetic effects similar to those of H_2_O_2_, which were inhibited by the antioxidant *N*-acetylcysteine. Oxidant-induced bioenergetic changes, loss of ΔΨ_m_, and cell death were not ameliorated by genetic deletion of CypD or by its acute inhibition with cyclosporine A. These results indicate that oxidative stress alters mitochondrial bioenergetics and modifies pancreatic acinar cell death. A shift from apoptosis to necrosis appears to be associated with decreased mitochondrial spare respiratory capacity and ATP production, effects that are independent of CypD-sensitive MPTP formation.

## Introduction

Acute pancreatitis (AP)^3^ is a severe inflammatory disorder, triggered primarily by excessive gallstones or excessive alcohol consumption, that can lead to a systemic inflammatory response syndrome, multiple organ failure, and death of the patient (1). It is one of the most common causes of emergency hospital admission from gastrointestinal causes in the United States, with an annual cost of U$ 2.6 billion (2). However, the underlying pathophysiology of AP is incompletely understood, and currently there is no specific therapy (3). The initial focus of damage is considered to be the pancreatic acinar cell, which manifests pathological changes including premature protease activation, vacuolization, and necrotic cell death pathway activation (4). Diverse AP precipitants have been shown to induce Ca^2+^-dependent mitochondrial depolarization, loss of ATP production, and acinar cell necrosis. Recent studies have demonstrated the central role of mitochondrial permeability transition pore (MPTP) formation in AP (5, 6), a Ca^2+^-sensitive channel modulated by cyclophilin D that allows movement of solutes <1.5 kDa in and out of the mitochondria (7). Although elevation of mitochondrial matrix Ca^2+^ is the principal trigger for MPTP formation, oxidative stress has been implicated in pore opening (7, 8). For example, fibroblasts and hepatocytes from mice lacking CypD (*Ppif*^−/−^) were partially protected from H_2_O_2_-induced cell death (9–11), although whether the MPTP is modulated by oxidative stress in the pancreas is unclear.

Oxidative stress is a prominent feature of AP in preclinical and clinical studies (12). Increases of reactive oxygen species (ROS) and their by-products were detected in patients, concurrent with a suppression of antioxidant defenses, which correlated with disease severity (13, 14). However, the precise role of ROS in pancreatic pathophysiology remains unclear and clinical trials of antioxidant therapy have demonstrated no clear benefit in the treatment of AP (12). We have shown that generation of ROS may constitute a protective mechanism that disposes of stressed pancreatic acinar cells, because bile acid-induced ROS production increased apoptosis with a concomitant reduction of necrosis (15). The extent of pancreatic necrosis is linked to more severe clinical disease (16), and the apoptosis/necrosis balance may therefore be an important determinant of AP progression.

The aim of the present study was to determine the effects of oxidants on pancreatic acinar cell bioenergetics, mitochondrial dysfunction, and cell death using multiple approaches; the role of CypD was evaluated using a knockout mouse model (*Ppif*^−/−^) and pharmacological inhibition. Our data demonstrate that the level of oxidative stress applied modified mitochondrial bioenergetics and determined cell death patterns independently of CypD-sensitive MPTP formation.

## Results

### Concentration-dependent inhibitory effects of H_2_O_2_ on apoptotic and necrotic cell death pathway activation

**Figure 1.**
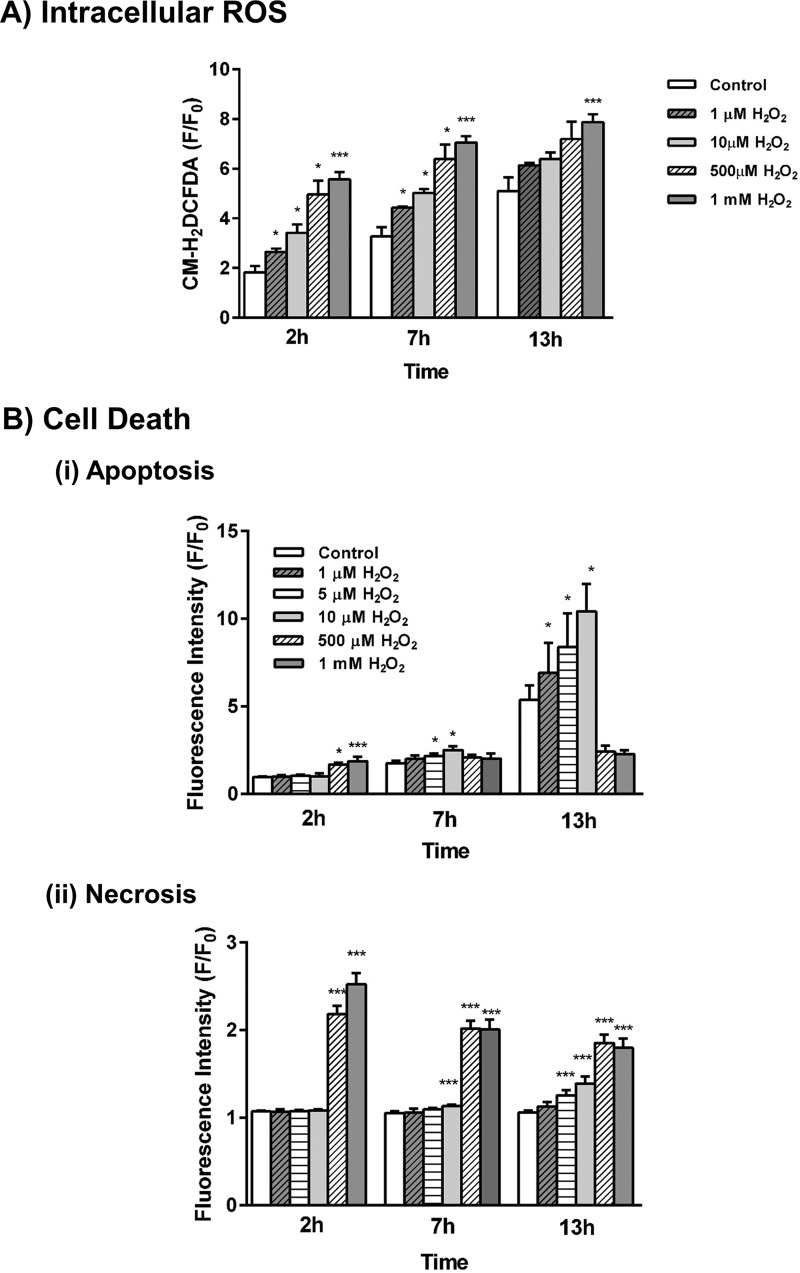
**Effects of H_2_O_2_ on intracellular ROS levels and cell death.** Concentration-dependent effects of H_2_O_2_ (1 μm to 1 mm) on intracellular ROS levels (*A*, chloromethyl 2′,7′-dichlorodihydrofluorescein diacetate (CM-H_2_DCFDA), apoptosis (*B*, *panel i*, caspase-3/7, *green*), and necrosis (*B*, *panel ii*, propidium iodide) in isolated murine pancreatic acinar cells measured over a 13-h period (H_2_O_2_ was applied at time 0). The changes are normalized increases in fluorescence from the baseline (*F*/*F*_0_) and expressed as the means ± S.E. (*n* = 6). Significant differences from the control are shown as follows: *, *p* < 0.05; **, *p* < 0.01; and ***, *p* < 0.001.

Application of H_2_O_2_ (1 μm to 1 mm) to pancreatic acinar cells caused a time-dependent increase of intracellular ROS that attained a maximal response at 500 μm (Fig. 1*A*). The characteristics of the response varied with the H_2_O_2_ concentration applied; rapid elevations were detected in pancreatic acinar cells at higher (500 μm to 1 mm) H_2_O_2_ levels, whereas lower levels (1–10 μm) induced more slowly developing rises. Accordingly, stimulation of acinar cells with H_2_O_2_ elicited cell death pathway activation that varied according to the severity of insult applied. Lower concentrations (1–10 μm) of H_2_O_2_ preferentially promoted time- and concentration-dependent apoptotic cell death pathway activation, with relatively little induction of necrosis until later time points (Fig. 1*B*). Conversely, higher H_2_O_2_ concentrations (500 μm to 1 mm) induced rapid necrosis with minimal transient elevation of apoptotic cell death; necrotic cell death exceeded twice the control value at 2 h and was sustained over the experimental period (Fig. 1*B*).

### Concentration-dependent inhibitory effects of H_2_O_2_ on the redox ratio (NADH/FAD^+^) and mitochondrial membrane potential (ΔΨ_m_)

**Figure 2.**
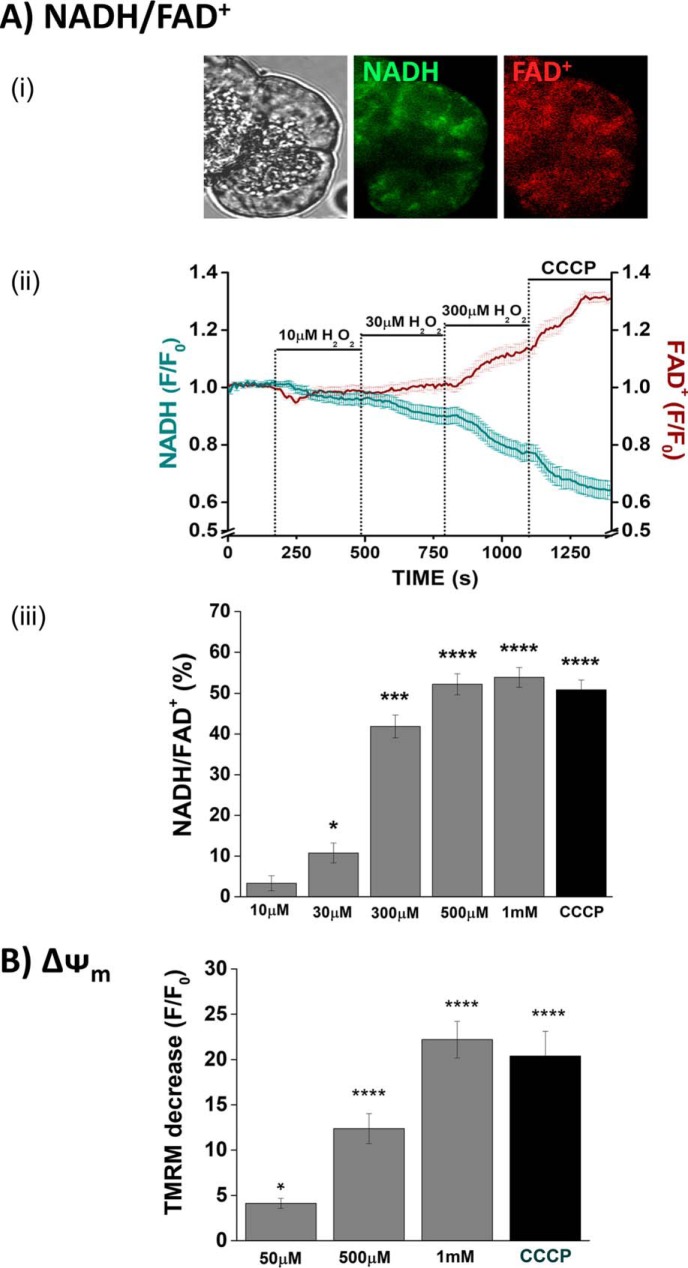
**Effects of H_2_O_2_ on redox ratio and mitochondrial membrane potential.** *A*, *panel i*, typical images showing the transmitted light (*left panel*) and mitochondrial localization of NADH and FAD^+^ autofluorescence in a pancreatic acinar cell, measured simultaneously using confocal microscopy. *Panel ii*, concentration-dependent effects of H_2_O_2_ (10–300 μm) and CCCP on NADH and FAD^+^ levels, expressed as normalized values from control (*F*/*F*_0_). *Panel iii*, effects of H_2_O_2_ (10 μm to 1 mm) on the redox ratio (NADH/FAD^+^) expressed as percentages of basal values; CCCP applied to show a maximal effect. *B*, effects of H_2_O_2_ on mitochondrial membrane potential (ΔΨ_m_), expressed as the percentages of decrease of TMRM fluorescence (averages of ≥56 cells from >4 animals). The data have been normalized to the initial fluorescence at *t* = 0 (*F*/*F*_0_). All data shown are the means ± S.E. Significant differences from the control are shown as follows: *, *p* < 0.05; **, *p* < 0.01; ***, *p* < 0.001; and ****, *p* < 0.0001.

In confocal microscopy experiments cellular NADH and FAD^+^ autofluorescence was distributed in a typical mitochondrial arrangement, as described previously in isolated pancreatic acinar cells (17) (Fig. 2*A*, *panel i*). Application of H_2_O_2_ (10 μm–1 mm) induced concentration-dependent decreases of NADH, effects mirrored by increases of FAD^+^ (Fig. 2*A*, *panel ii*). These bioenergetic changes were maximal at 500 μm; subsequent application of the protonophore CCCP (10 μm) produced no further changes in the NADH/FAD^+^ ratio (Fig. 2*A*, *panel iii*). In separate experiments, H_2_O_2_ at concentrations of ≥50 μm induced a concentration-dependent diminution of the mitochondrial membrane potential that was maximal at the highest concentration of H_2_O_2_ (1 mm). The addition of CCCP induced no further depolarization (Fig. 2*B*).

### Concentration-dependent inhibitory effects of H_2_O_2_ and menadione on acinar cell bioenergetics

Seahorse flux analysis showed that freshly isolated pancreatic acinar cells exhibited a basal O_2_ consumption rate (OCR) of 462.24 ± 28.78 pmol/min, (*n* = 10 independent experiments); this was ∼56% of the maximal oxygen consumption achievable, measured by use of the uncoupling agent FCCP. This finding indicates the presence of a significant reserve (spare respiratory capacity) in this cell type that is available when bioenergetic demand is increased. The pancreatic acinar cells exhibited a basal extracellular acidification rate (ECAR) of 10.81 ± 0.91 mpH/min, an indirect index of glycolysis reflecting cellular lactate production (*n* = 10 independent experiments).

**Figure 3.**
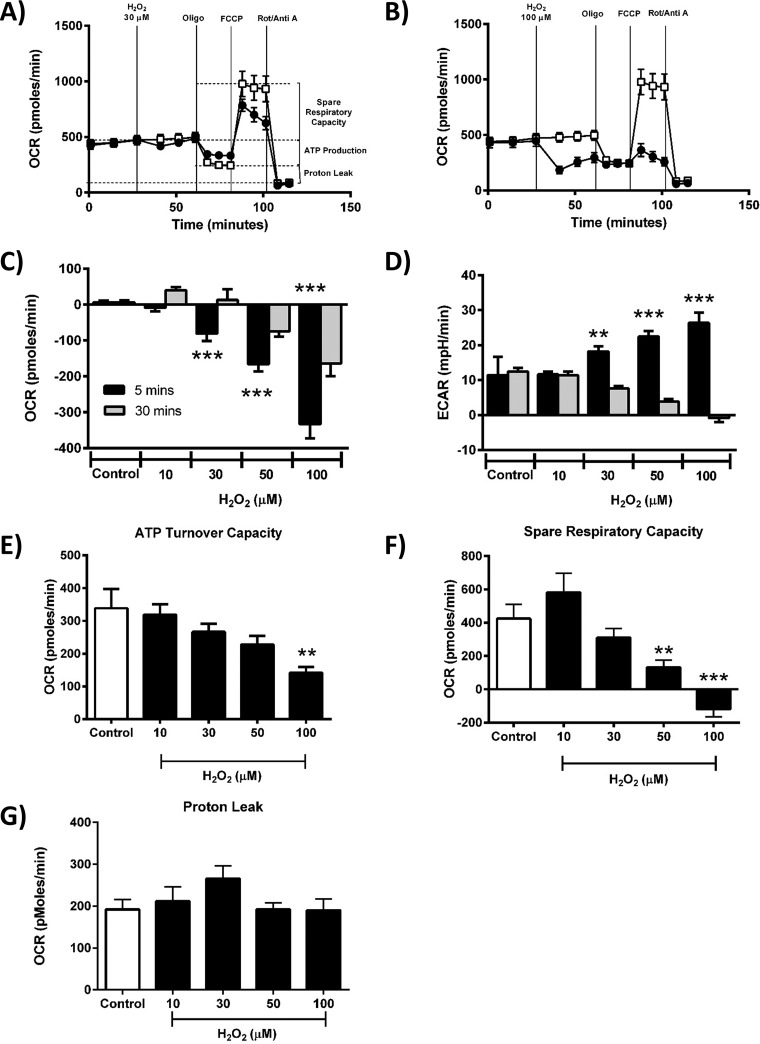
**Effects of H_2_O_2_ on mitochondrial bioenergetics.** *A* and *B*, the effects of H_2_O_2_ (●) at 30 μm (*A*) and 100 μm (*B*) on the OCR in isolated pancreatic acinar cells compared with controls without H_2_O_2_ addition (□). *C–G*, a respiratory function “stress” test was carried out using sequential addition of oligomycin (*Oligo*, 1 μg/ml), FCCP (0.3 μm), and rotenone (*Rot*)/antimycin A (*Anti A*) combined (1 μm) injected sequentially. Acute effects of H_2_O_2_ (10–100 μm) on baseline changes of OCR (*C*) and ECAR (*D*) after 5 min (*black*) and 30 min (gray), ATP turnover capacity (*E*), spare respiratory capacity (*F*), and proton leak (*G*). The data are shown as the means ± S.E. (*n* = 3). Significant differences from the control are shown as follows: *, *p* < 0.05; **, *p* < 0.01; and ***, *p* < 0.001.

Application of H_2_O_2_ (30–100 μm) caused a concentration-dependent decrease in basal respiration, an effect commencing within 5 min of application that was sustained over a 30-min period (Fig. 3, *A–C*). This depression of OCR was accompanied by a concentration-dependent increase in ECAR within 5 min; however, this elevation was not sustained over 30 min (Fig. 3*D*). Use of the respiratory function (“stress”) test, requiring sequential addition of inhibitors of the electron transport chain, revealed the inhibitory effects of H_2_O_2_ on mitochondrial bioenergetics. The mitochondrial ATP turnover capacity, visualized after addition of oligomycin, was concentration-dependently decreased by H_2_O_2_ (Fig. 3*E*). At concentrations of ≥50 μm H_2_O_2_ significantly reduced the spare respiratory capacity (Fig. 3*F*), with no effect on proton leak (Fig. 3*G*).

**Figure 4.**
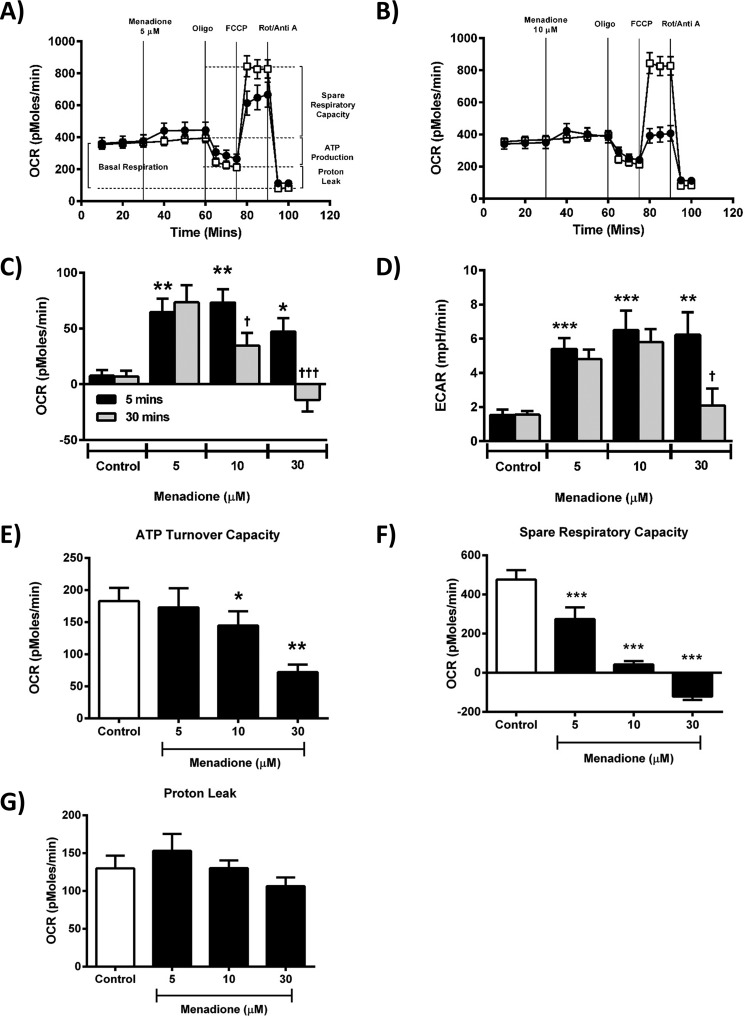
**Effects of menadione on mitochondrial bioenergetics.** *A* and *B*, the effects of menadione (●) at 5 μm (*A*) and 10 μm (*B*) on the oxygen consumption rate (OCR) in isolated pancreatic acinar cells compared with controls without menadione addition (□). A respiratory function “stress” test was carried out using sequential addition of oligomycin (*Oligo*, 1 μg/ml), FCCP (0.3 μm), and rotenone (*Rot*)/antimycin A (*Anti A*) combined (1 μm) injected sequentially. *C–G*, acute effects of menadione (5–30 μm) on baseline changes of OCR (*C*) and ECAR (*D*) after 5 min (*black*) and 30 min (*gray*), ATP turnover capacity (*E*), spare respiratory capacity (*F*), and proton leak (*G*). The data are shown as the means ± S.E. (*n* = 8). Significant differences are shown as follows: *, *p* < 0.05; **, *p* < 0.01; and ***, *p* < 0.001 compared with control; and ^$^, *p* < 0.05; ^$$^, *p* < 0.01; and ^$$$^, *p* < 0.001 compared with the 5-min time point.

The oxidant menadione, which has been shown to generate ROS in pancreatic acinar cells via a redox cycle that consumes NADH (18), exerted predominantly inhibitory actions on mitochondrial bioenergetics. In common with H_2_O_2_, menadione (5–30 μm) greatly decreased the acinar cell spare respiratory capacity in a concentration-dependent manner with a maximal effect observed at 30 μm (Fig. 4, *A*, *B*, and *F*). At concentrations of ≥10 μm, menadione also decreased ATP turnover capacity (Fig. 4*E*). However, in contrast to the suppression of basal respiration observed with H_2_O_2_, menadione caused a slight increase of OCR *per se*, an effect that gradually declined over the 30-min application. At the highest concentration tested (30 μm), menadione caused a significant decrease in basal respiration compared with the control at 30 min (Fig. 4, *A–C*). In common with H_2_O_2_, menadione induced a significant elevation of ECAR at all concentrations within 5 min (Fig. 4*D*), an effect maintained at lower concentrations throughout the application.

### Inhibitory effects of H_2_O_2_ and menadione on bioenergetics reduce ATP production and cell viability

**Figure 5.**
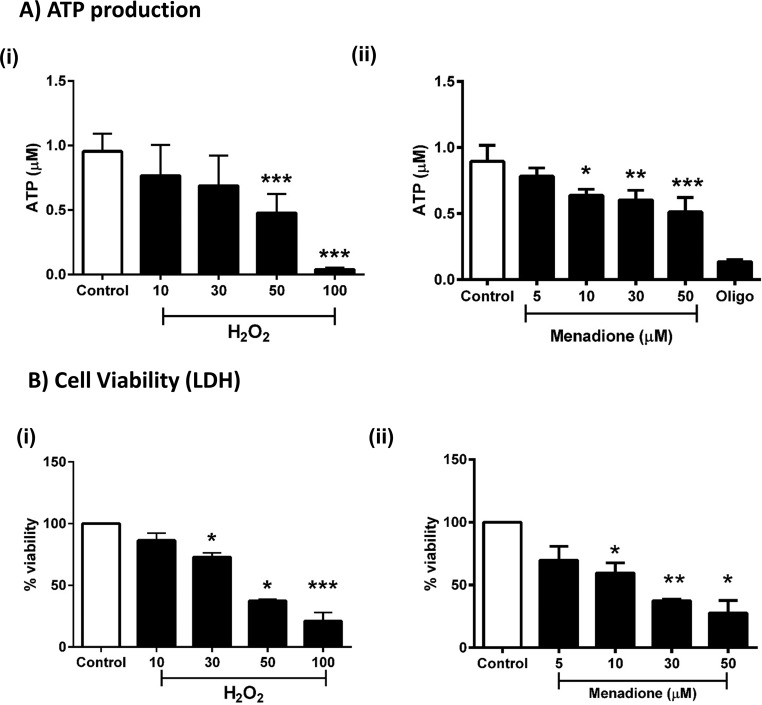
**Effects of H_2_O_2_ and menadione on ATP levels and cell viability.** *A* and *B*, the effects of H_2_O_2_ (10–100 μm) and menadione (5–50 μm) on ATP levels measured via luciferase assay (*A*) and cell viability (lactate dehydrogenase levels (*LDH*)) (*B*). Oligomycin (*Oligo*, 1 μg/ml) was added to show a maximal effect in *A*, *panel ii*. The data are shown as the percentages of viability, expressed as the means ± S.E. (*n* = 3). Significant differences from the control are shown as follows: *, *p* < 0.05; **, *p* < 0.01; and ***, *p* < 0.001.

To determine whether the detrimental changes of bioenergetics induced by oxidants in acinar cells resulted in a reduction of ATP levels, separate luciferase-based plate-reader assays were conducted. Application of H_2_O_2_ (10–100 μm) and menadione (5–50 μm) to pancreatic acinar cells caused concentration-dependent decreases of cellular ATP (Fig. 5*A*, *panels i* and *ii*). The addition of the ATP synthase inhibitor oligomycin was used to show maximal blockade of mitochondrial respiration (Fig. 5*A*, *panel ii*). Application of H_2_O_2_ and menadione in separate plate-reader assays induced concentration-dependent decreases of pancreatic acinar cell viability at concentrations of ≥30 μm and ≥10 μm, respectively (Fig. 5*B*, *panels i* and *ii*).

### Oxidant-induced bioenergetic changes and cell death are inhibited by N-acetylcysteine

**Figure 6.**
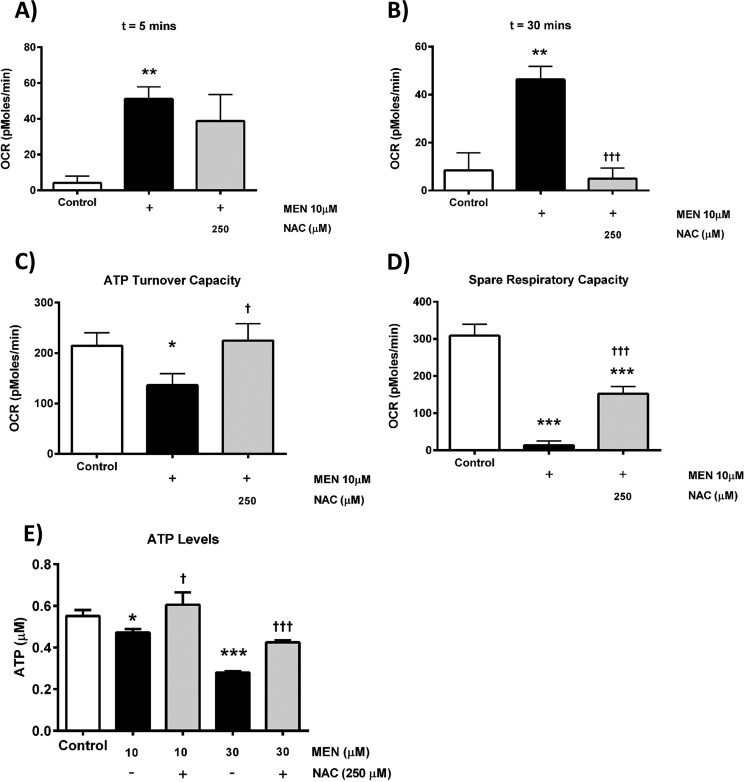
**Effects of *N*-acetylcysteine on menadione-induced mitochondrial bioenergetic inhibition.** *A–D*, the effects of NAC (250 μm) on menadione-induced (*MEN*, 10 μm) changes of the OCR in isolated pancreatic acinar cells after 5 min (*A*) and after 30 min (*B*), ATP turnover capacity (*C*), and spare respiratory capacity (*D*, *n* = 3). *E*, the effects of NAC (250 μm) on menadione-induced (10 and 30 μm) reductions of ATP levels measured via luciferase assay. The data are shown as the means ± S.E. (*n* = 4). Significant differences from the control are shown as follows: *, *p* < 0.05; **, *p* < 0.01; and ***, *p* < 0.001 compared with control; and †, *p* < 0.05; ††, *p* < 0.01; and †††, *p* < 0.001 compared with menadione alone.

**Figure 7.**
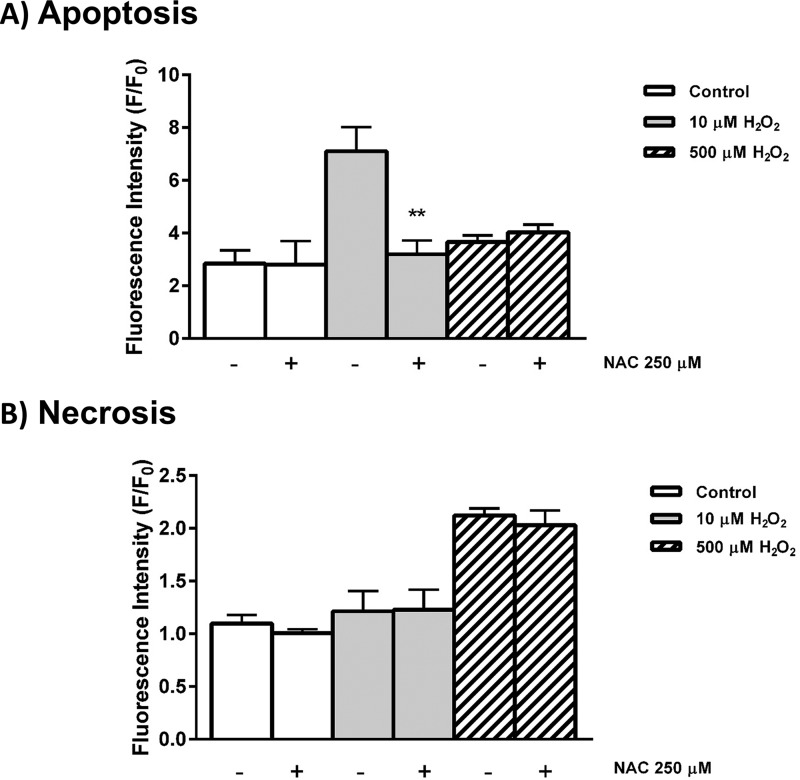
**Effects of NAC on H_2_O_2_-induced cell death.** The effects of 250 μm NAC are shown on the concentration-dependent actions of H_2_O_2_ (10 and 500 μm) on apoptosis (*A*, caspase-3/7, *green*) and necrosis (*B*, propidium iodide) in isolated murine pancreatic acinar cells measured at 13 h (H_2_O_2_ was applied at time 0). The changes are normalized increases of fluorescence from the baseline (*F*/*F*_0_) and expressed as the means ± S.E. (*n* = 3). Significant differences from the control are shown as follows: *, *p* < 0.05; **, *p* < 0.01; and ***, *p* < 0.001.

To confirm that the observed actions of exogenously applied oxidants on cellular bioenergetics and fate were mediated through ROS generation, the effects of *N*-acetylcysteine (NAC; 250 μm) on menadione-induced changes on OCR were assessed. NAC inhibited the stimulation of basal respiration elicited by 10 μm menadione (Fig. 6, *A* and *B*) and increase in ECAR (data not shown). Similarly, NAC application significantly inhibited oxidant-induced decrease of ATP turnover and spare respiratory capacity. Furthermore, the reductions of cellular ATP measured by luciferase-based plate-reader assay induced by menadione were significantly inhibited by 250 μm NAC (Fig. 6*E*). In separate experiments, NAC (250 μm) reversed the increase of apoptosis induced by low (10 μm) H_2_O_2_ back to basal levels but did not prevent the necrosis caused by high (500 μm) H_2_O_2_ (Fig. 7).

### H_2_O_2_- and menadione-induced effects on bioenergetics, ΔΨ_m_, and cell death are MPTP-independent

**Figure 8.**
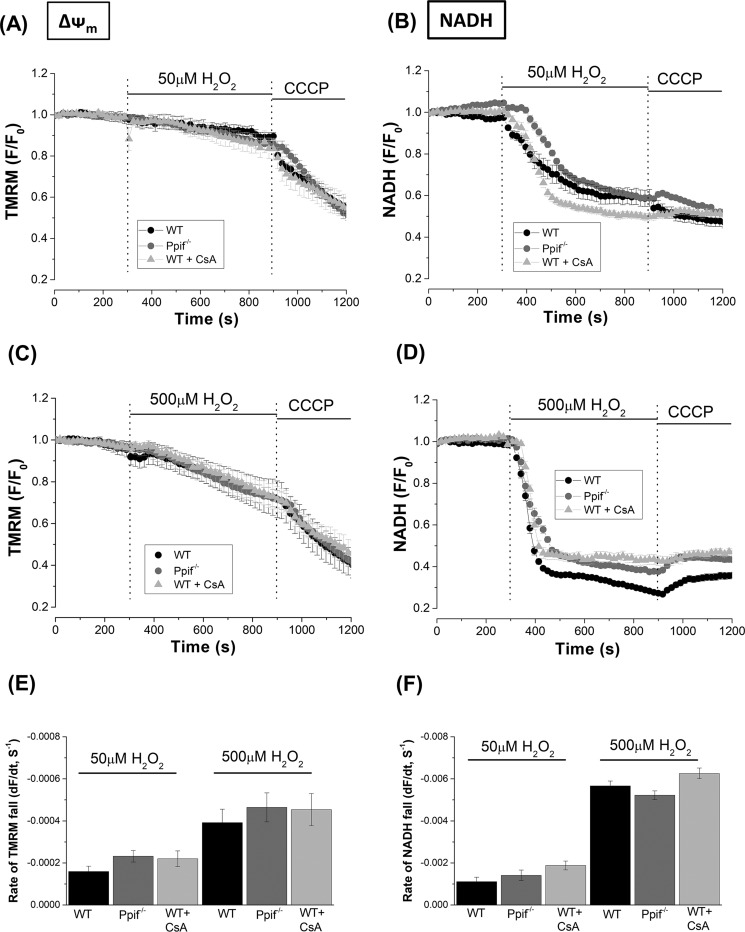
**Effects of cyclophilin D knockout (*Ppif*^−/−^) and pharmacological inhibition on H_2_O_2_-induced mitochondrial depolarization and reduction of NADH.** *A–D*, the effects of 50 μm (*A* and *B*) and 500 μm (*C* and *D*) H_2_O_2_ on mitochondrial membrane potential (ΔΨ_m_) and NADH autofluorescence in pancreatic acinar cells isolated from *Ppif*^−/−^ (*gray*) and WT (C57Bl6, *black*) mice. The effects of cyclosporine A (CsA) treatment on changes in WT to H_2_O_2_ are also shown. Changes are expressed as normalized decreases of TMRM fluorescence and falls of NADH from basal levels (*F*/*F*_0_); the mitochondrial uncoupler CCCP (10 μm) was applied to show maximal depolarization. *E* and *F*, the concentration-dependent rates of fall of TMRM fluorescence and NADH autofluorescence in response to H_2_O_2_ under the various conditions are displayed. All data are shown as the means ± S.E. (averages of ≥80 cells from >4 animals).

ROS may be an important trigger for MPTP formation in excitable tissues including cardiac muscle (19) and neurones (20); therefore possible involvement of the pore in pancreatic acinar cells was assessed by comparing the effects of H_2_O_2_ on bioenergetics and cell death in cyclophilin D knockout (*Ppif*^−/−^) and WT (C57BL6) mice. In confocal microscopy experiments, application of H_2_O_2_ (50 and 500 μm) induced concentration-dependent decreases of NADH and ΔΨ_m_ in pancreatic acinar cells (Fig. 8, *A–D*). These responses were not significantly altered by genetic deletion of CypD. Furthermore, acute inhibition of CypD with cyclosporine A (CsA) did not prevent the effects of H_2_O_2_ (Fig. 8).

**Figure 9.**
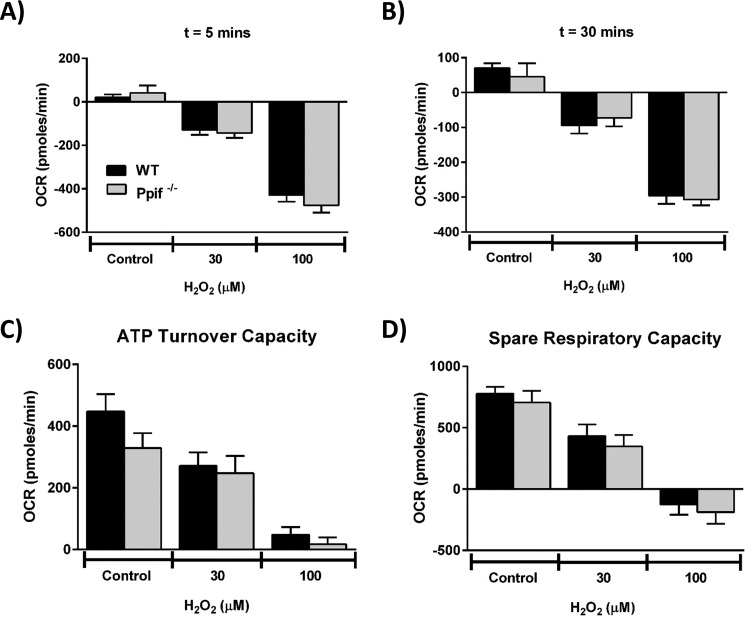
**Effects of cyclophilin D knockout (*Ppif*^−/−^) on H_2_O_2_-induced changes of mitochondrial bioenergetics.** The effects of H_2_O_2_ (30 and 100 μm) on the OCR in pancreatic acinar cells isolated from *Ppif*^−/−^ (*gray*) and WT (C57Bl6, *black*) mice after 5 min (*A*) and after 30 min (*B*), ATP turnover capacity (*C*), and spare respiratory capacity (*D*). The data are shown as the means ± S.E. (*n* = 3).

**Figure 10.**
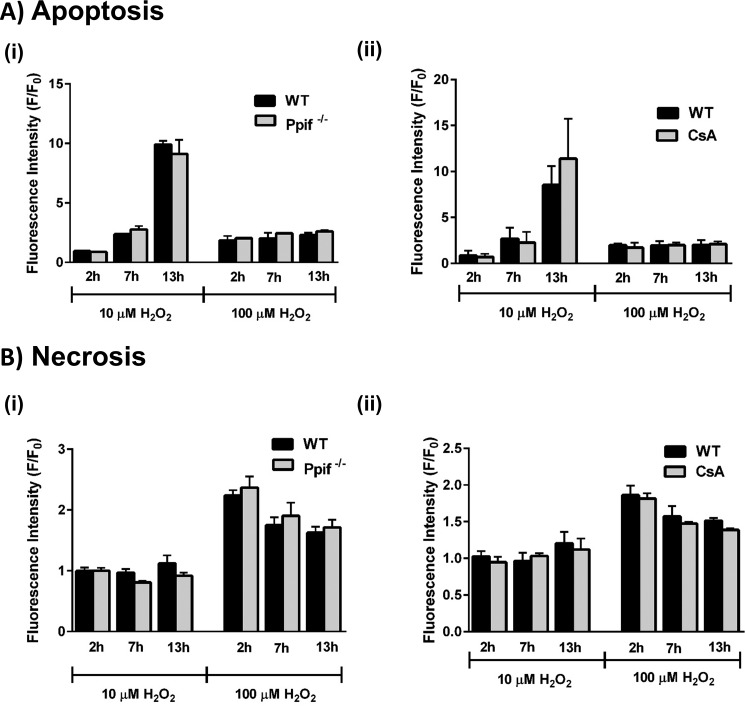
**Effects of cyclophilin D knockout (*Ppif*^−/−^) and pharmacological inhibition on H_2_O_2_-induced cell death.** *A* and *B*, the effects of H_2_O_2_ (10 and 100 μm) on apoptosis (*A*, caspase 3/7, *green*) and necrosis (*B*, propidium iodide) in pancreatic acinar cells isolated from *Ppif*^−/−^ (*gray*) and WT (C57Bl6, *black*) mice (*panel i*) and in WT (*black*) and CsA-treated WT (*gray*) (*panel ii*). The data were normalized to the initial fluorescence at time 0 (*F*/*F*_0_) and expressed as the means ± S.E. (*n* = 6).

Bioenergetics evaluations using the Seahorse XF analyzer demonstrated that the detrimental changes on respiration induced by H_2_O_2_ (10 and 30 μm) were not significantly different between *Ppif*^−/−^ and WT mice (Fig. 9). Thus, the concentration-dependent decreases of basal respiration, ATP turnover, and spare respiratory capacity were unaffected by the absence of CypD. Similarly, the profile of cell death induced by H_2_O_2_ (10 and 100 μm) was not significantly different between *Ppif*^−/−^ and WT mice; apoptotic cell death, preferentially promoted by lower (10 μm) H_2_O_2_, and necrotic cell death by higher (100 μm) H_2_O_2_, was similar in the presence and absence of CypD (Fig. 10*A*, *panel i*, and *B*, *panel i*). Furthermore, apoptotic and necrotic pancreatic acinar cell death in WT mice were not inhibited by pharmacological inhibition of CypD with CsA (Fig. 10*A*, *panel ii*, and *B*, *panel ii*, respectively).

**Figure 11.**
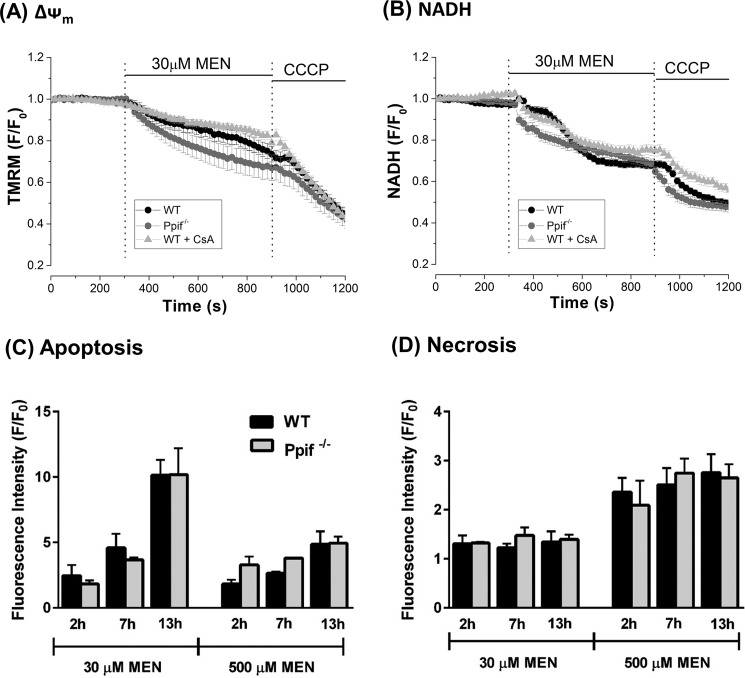
**Effects of cyclophilin D knockout (*Ppif*^−/−^) and pharmacological inhibition on menadione-induced mitochondrial depolarization, reduction of NADH, and pancreatic acinar cell death.** *A* and *B*, the effects of menadione (*MEN*, 30 μm) on mitochondrial membrane potential (*A*, ΔΨ_m_) and NADH autofluorescence (*B*) in pancreatic acinar cells isolated from *Ppif*^−/−^ (*gray*) and WT (C57Bl6, *black*), and in WT with CsA treatment. The changes are expressed as normalized decreases of TMRM fluorescence and falls of NADH from basal levels (*F*/*F*_0_); the mitochondrial uncoupler CCCP (10 μm) was applied to show maximal depolarization. *C* and *D*, the effects of menadione (30 and 500 μm) on apoptosis (*C*, caspase 3/7, *green*) and necrosis (*D*, propidium iodide) in pancreatic acinar cells isolated from *Ppif*^−/−^ (*gray*) and WT (9C57Bl6, *black*) mice. All data are shown as the means ± S.E. (*n* ≥ 3).

In separate experiments, the addition of menadione (30 μm) caused mitochondrial depolarization and a reduction of NADH in pancreatic acinar cells that was not inhibited by genetic deletion of CypD or acute inhibition with CsA (Figs. 11, *A* and *B*). Similarly, patterns of cell death evoked by the oxidant were not significantly different between WT and *Ppif*^−/−^ mice (Fig. 11, *C* and *D*).

## Discussion

Our results demonstrate that oxidants affected pancreatic acinar cell bioenergetics and fate independently of CypD-sensitive MPTP formation. The precise composition of the MPTP is currently unknown and controversial, with recent evidence suggesting that it may comprise a dimer of the ATP synthase (21–23). However, other studies have cast doubt this, including evidence that deletion of ATP synthase subunits did not prevent permeability transition (24–26), and there is no consensus. Although the primary trigger for MPTP formation is high mitochondrial matrix Ca^2+^, oxidative stress has also been implicated (7, 8); it has been suggested that CypD may act as a redox sensor (27) and/or that the redox environment may directly gate the voltage sensor of the pore (28). Because the MPTP can be formed independently of CypD, this protein appears modulatory rather than a core structural component (29, 30), with CypD-deficient mitochondria able to undergo permeability transition, albeit with a decreased sensitivity to Ca^2+^ (7, 9, 31).

Few investigations, however, have used genetic deletion of *Ppif* to specifically evaluate whether CypD mediates ROS-induced MPTP formation. Studies have shown that cultured fibroblasts and hepatocytes from *Ppif*^−/−^ mice were partially protected from Ca^2+^ overload and oxidative stress-induced cell death (9–11). In contrast, CypD deletion affected the sensitivity of the MPTP in primary hepatocytes to Ca^2+^ but not to oxidative stress, with mitochondria from *Ppif*^−/−^ mice as sensitive to the MPTP-inducing effects of thiol oxidants as WT animals (31). Our current results clearly demonstrate that oxidant effects in exocrine pancreas did not involve CypD-dependent MPTP opening, because bioenergetic changes, ΔΨ_m_, and cell death were not different between *Ppif*^−/−^ and WT mice. Furthermore, the CypD inhibitor CsA, previously shown to protect against acinar cell necrosis induced by AP precipitants (5), did not prevent oxidant-induced bioenergetics changes and associated cell death. Our results in pancreatic acinar cells are therefore in agreement with the view that CypD modulates the sensitivity of the MPTP to Ca^2+^ but not to oxidative stress (31). A study using pancreatic acinar cells from Sprague-Dawley rats, however, has previously shown sensitivity of H_2_O_2_-induced mitochondrial depolarization to CsA, although a genetic deletion of CypD was not assessed (32); an apparent discrepancy with our current results may reflect species variation. A CypD-independent opening of the MPTP has been shown in isolated rat liver mitochondria and in CEM and HL60 cells (33); increased production of ROS generated by cytochrome *bc*_1_ activated a CsA-insensitive MPTP, whereas Ca^2+^-induced MPTP opening was inhibited by the CypD inhibitor. The existence of both regulated (Ca^2+^-activated and CsA-sensitive) and unregulated (CsA-insensitive) MPTP has previously been proposed (34, 35).

A pivotal role of MPTP formation has been demonstrated in mediating pancreatic necrosis during AP (5, 6). Inhibition of CypD, by both genetic knockout and pharmacological inhibition, was protective in multiple *in vivo* AP models and of human and murine pancreatic acinar necrosis *in vitro*. In contrast, apoptotic cell death was unaffected by CypD inhibition, consistent with previous findings in liver (11) and eosinophils (36), showing that necrotic but not apoptotic cell death was MPTP-dependent (11). Previously, MPTP-dependent pancreatic acinar cell death induced by menadione was reported (37), although no genetic knockout model was used, and conclusions were based solely on the effects of bongkrekic acid, an inhibitor of the adenine nucleotide transporter (ANT). It was subsequently demonstrated that the ANT is not a core MPTP component because mitochondria lacking ANT were capable of permeability transition, whereas hepatocytes deficient in Ant1 and Ant2 exhibited enhanced Ca^2+^-induced mitochondrial swelling and cell death (38). However, the previous study in acinar cells (37) and our current data may point to a CypD-independent mechanism of MPTP formation in the exocrine pancreas. Although the basis is currently unclear, direct lipid peroxidation and/or formation of products such as 4-hydroxynonenal known to damage cellular membranes may contribute to the progressive inhibition of bioenergetics caused by oxidants in the present study. However, no significant increase of proton leak was detected in response to either H_2_O_2_ or menadione, and more specific actions of ROS may be involved. Interestingly, elevation of matrix ROS has also been shown to activate UCP-2 (uncoupling protein 2) in kidney mitochondria (39), with up-regulation of UCP-2 reported in AP models that correlated with disease severity (40).

Importantly, the level of oxidative stress applied determined the bioenergetic profile and pattern of acinar cell death; at low oxidant concentrations, there was a preferential induction of apoptotic cell death, whereas higher levels rapidly caused necrosis. Our findings in primary cells are therefore consistent with studies showing that such oxidants exerted differential effects on apoptosis and necrosis in cultured T-lymphoma Jurkat cells (41) and AR42J cells (42), and strongly indicate an important role of redox status in determining pancreatic acinar cell death patterns. We have previously shown that mitochondrial ROS generation by menadione and bile acid promoted apoptotic death in human and murine pancreatic acinar cells by Ca^2+^-independent and Ca^2+^-dependent mechanisms, respectively (15, 18). Manipulation of ROS levels affected the apoptosis-necrosis balance; scavenging of ROS caused a relative increase in necrosis and decrease in apoptosis, whereas potentiation of ROS elicited the converse (15). Because the extent of necrosis determines the severity of experimental AP, induction of apoptosis by ROS may be beneficial (3, 43, 44). Under low stress conditions, in which slight elevations of ROS are associated with relatively mild bioenergetic inhibition, preferential promotion of apoptosis may be an efficient means of disposing of compromised acinar cells without instigation of necrosis that drives inflammation.

The likely trigger for change in cell death modality was inhibition of mitochondrial ATP production (45) because the shift from apoptotic to necrotic cell death at higher oxidant concentrations was coincident with a marked diminution of ATP turnover. Interestingly, in contrast to H_2_O_2_, which acutely inhibited basal respiration, menadione caused a transient elevation of OCR before inhibitory effects ensued. Unlike H_2_O_2_, quinones undergo fast redox cycles that generate ROS and consume NAD(P)H (18); raised OCR may therefore indicate a compensatory boost of metabolism as NADH is used. However, the predominant action of ROS elevation by both oxidants was to depress mitochondrial bioenergetics. Mitochondrial dysfunction is a central feature of AP pathophysiology (3, 46), with diverse AP precipitants inducing sustained elevations of cytosolic and mitochondrial Ca^2+^ (15, 47–49) that compromise ATP production (47, 50). Our study has demonstrated that the spare respiratory capacity in acinar cells was progressively diminished as the level of oxidative stress increased, an action linked to reduced cell viability. This important bioenergetic parameter is decreased under pathophysiological conditions, including cardiac and neurodegenerative damage (51–53), and indicates an insufficiency of the acinar cell to meet its metabolic demands under conditions of elevated stress. In addition, the action of oxidants on respiration was associated with a boost of glycolysis, potentially as a compensatory mechanism to maintain cellular ATP. Nevertheless, the fall in cellular ATP observed at higher oxidant levels would compromise caspase activation integral to execution of apoptosis; caspase inhibition exacerbated pancreatic acinar cell necrosis and worsened experimental AP (54). Other mechanisms may also contribute to cell death patterns because caspases are directly regulated by the cellular redox state. Thus, in Jurkat cells caspase-3 activity was differentially regulated according to the extent of H_2_O_2_-induced ROS generation, with lower levels increasing activity, whereas higher concentrations were inhibitory (55). Consequently, an apoptotic phenotype would be compatible with low oxidative stress, whereas high ROS levels would shift cell death toward necrosis (56). Consistent with our present findings, H_2_O_2_ inhibited plasmalemmal Ca^2+^-ATPase in pancreatic acinar cells at concentrations above 50 μm (32), implying a role for excessive oxidative stress in the Ca^2+^ overload that drives necrosis (57).

In conclusion, our results demonstrate that oxidants altered mitochondrial bioenergetics in pancreatic acinar cells and modified cell fate, resulting in a shift from apoptosis to necrosis, independently of CypD-sensitive MPTP formation. Because oxidative stress is a feature of clinical acute pancreatitis, it is likely to exert an important influence on local cell death patterns underlying disease pathophysiology and to be an important factor with respect to potential therapeutic intervention via MPTP inhibition (5, 58).

## Experimental procedures

### Pancreatic acinar cell preparation and solutions

Cyclophilin d-deficient mice were generated by targeted disruption of the *Ppif* gene and generously provided by Dr. D. Yellon (University College, London, UK) and Dr. M. A. Forte (Oregon Health and Sciences University). Genotyping of mice was performed using standard PCR with a specific primer set (Exon 3-F, CTC TTC TGG GCA AGA ATT GC; Neo-F, GGC TGC TAA AGC GCA TGC TCC; and Exon 4-R, ATT GTG GTT GGT GAA GTC GCC) to confirm deletion of CypD (Fig. S1*A*). Furthermore, mitochondria isolated from these animals showed a typical resistance to MPTP formation in a classical Ca^2+^ retention assay (Fig. S1*B*). Fresh pancreatic acinar cells were isolated using standard collagenase (Worthington Biochemical Corporation, Lakewood, NJ) from pancreata of young (8–12 week old) adult C57BL/6 (WT) and *Ppif*^−/−^ mice as previously described (5, 15). The animals were humanely sacrificed by cervical dislocation (schedule 1 procedure) in accordance with the Animals (Scientific Procedures) Act (1986) under Establishment License 40/2408 and with approval by the University of Liverpool Animal Welfare Committee and ethical review body. The extracellular solution contained 140 mm NaCl, 4.7 mm KCl, 1.13 mm MgCl_2_, 1 mm CaCl_2_, 10 mm d-glucose, and 10 mm HEPES (adjusted to pH 7.25 using NaOH). H_2_O_2_ and menadione (Sigma) were applied to pancreatic acinar cells to induce oxidative stress, and *N*-acetylcysteine (Sigma) was utilized as an antioxidant.

### Confocal microscopy

Confocal imaging was performed using a Zeiss LSM510 system (Carl Zeiss). Freshly dispersed acinar cells were loaded with 37 nm tetramethyl rhodamine methyl ester (TMRM; excitation, 543 nm; and emission, 560–650 nm) for 30 min at room temperature for mitochondrial membrane potential (ΔΨ_m_) measurements. Mitochondrial metabolism was assessed in unloaded cells by NADH (excitation, 363 nm; and emission, 390–450 nm) and FAD^+^ (excitation, 458 nm; and emission, 505–560 nm) autofluorescence simultaneously. The redox ratio was determined by calculating the ratio of the measured fluorescence intensities of NADH and FAD^+^ (59, 60). Fluorescence measurements are expressed as changes from basal fluorescence (*F*/*F*_0_ ratio), where *F*_0_ represents the initial fluorescence recorded at the start of the experiment, and *F* represents the fluorescence recorded at specific time points (*n* represents the number of cells studied for each experimental protocol).

### Detection of reactive oxygen species

For ROS measurement isolated pancreatic acinar cells were loaded with chloromethyl 2′,7′-dichlorodihydrofluorescein diacetate (CM-H_2_DCFDA, 5 μm) for 30 min at 37 °C. The cells were plated at a density of 300,000/well and ROS detected using a POLARstar Omega plate reader (excitation, 488 nm; and emission, 520 nm; BMG Labtech). Triplicates were run for each condition, and fluorescence intensity was normalized to negative controls for each mouse.

### Detection of apoptotic and necrotic cell death pathways

For detection of necrosis and apoptosis, a POLARstar Omega fluorescence microplate reader (BMG Labtech) was employed for time-course experiments at 37 °C. Flat-bottomed 96-well microplates (Greiner Bio-One Ltd) were used to seed cells at a density of 300,000/well. Propidium iodide was used to detect necrosis and loaded at a final concentration of 10 μg/ml. Excitation was set at 520 nm, and emission collection was set at >590 nm. For apoptosis measurements, CellEvent® caspase-3/7 Green Ready® probes reagent was added to the acinar cell suspension at 40 μl/ml. Excitation was 485 nm, and emission was at 530 nm. The fluorescence intensity was normalized to negative controls for each mouse.

### Oxygen consumption and lactate production analysis

The XF24 analyzer (Seahorse Biosciences, North Billerica, MA) was used to measure bioenergetic function in pancreatic acinar cells. The XF24 measures OCR and ECAR in unloaded cells, monitored in real time. Prior to bioenergetic measurements, the isolation medium was changed to unbuffered DMEM (pH 7.4) supplemented with 10 mm glucose, 2 mm l-glutamine, and 2 mm sodium pyruvate (Sigma–Aldrich). The optimum number of cells/well for detection of changes in OCR and ECAR was determined to be 75,000/0.32 cm^2^. A mitochondrial respiratory function “stress” test protocol was implemented to measure indices of mitochondrial function with and without oxidative stress applied. Oligomycin, FCCP, antimycin A, and rotenone were injected sequentially through ports of the Seahorse Flux Pak cartridges to achieve final concentrations of 1, 76, and 2 μg/ml, respectively. Using these agents, the basal OCR, oxygen consumption linked to ATP production, level of non-ATP-linked oxygen consumption (proton leak), maximal and spare respiration capacity, and nonmitochondrial oxygen consumption were determined. Because H_2_O_2_ is an oxidant and may potentially interfere with the Seahorse O_2_ sensor, control experiments were performed in which H_2_O_2_ (10 μm to 1 mm) was added to empty wells and OCR measured; no changes were observed indicating suitability of this experimental protocol (Fig. S2).

### Intracellular ATP determination

Pancreatic acinar cells (1 × 10^6^/condition) were pretreated with oxidant for 30 min and washed with buffer A (25 mm Tris-HCl, 10 mm KH_2_PO_4_, 150 mm KCl, 5 mm MgCl_2_, 0.1% BSA, pH 7.8). The cells were then covered and permeabilized with 200 μl of 1× ATP-releasing reagent in buffer A (Sigma–Aldrich) per well for 2 min. Then 20 μl of each supernatant was transferred to a white plate, and the measurement protocol was started immediately using a POLARstar Omega plate reader (BMG Labtech). 80 μl of master mix, consisting of 0.3 mm luciferin potassium salt and luciferase (Sigma–Aldrich), was injected per well, and the luminescence emission was recorded for 15 min. The addition of the ATP synthase inhibitor oligomycin was used to show maximal blockade of mitochondrial respiration. The chemiluminescence intensity was normalized to negative controls for each mouse/run.

### Lactate dehydrogenase assay

Pancreatic acinar cells were pretreated with oxidant for 30 min, centrifuged and washed before using a lactate dehydrogenase activity assay kit (Sigma–Aldrich). The samples were added per well and the absorbance measurement was started immediately using a POLARstar Omega Plate Reader (BMG Labtech).

### Statistical analysis

Prism 5.0 software (GraphPad Software Inc., La Jolla, CA) was used to perform statistical analyses. All data were analyzed using analysis of variance and Tukey's post-test and are presented as means ± S.E. All experiments were repeated at least three times.

## Author contributions

J. A. A., N. J. C., Y. O., J. C. M., M. C., D. L., M. A., and D. N. C. data curation; J. A. A., N. J. C., Y. O., J. C. M., M. C., and D. N. C. formal analysis; J. A. A., N. J. C., Y. O., J. C. M., M. C., A. V. T., R. S., and D. N. C. investigation; J. A. A., N. J. C. methodology; J. A. A. and D. N. C. writing-original draft; M. C., A. V. T., R. S., and D. N. C. writing-review and editing; A. V. T., R. S., and D. N. C. funding acquisition; D. N. C. conceptualization; D. N. C. supervision; D. N. C. project administration.

## Supplementary Material

Supporting Information

## Footnotes

3 The abbreviations used are:

AP: acute pancreatitis
MPTP: mitochondrial permeability transition pore
CypD: cyclophilin D
ROS: reactive oxygen species
CCCP: carbonyl cyanide *p*-chlorophenylhydrazone
OCR: O_2_ consumption rate
FCCP: carbonyl cyanide *p*-trifluoromethoxyphenylhydrazone
ECAR: extracellular acidification rate
NAC: *N*-acetylcysteine
CsA: cyclosporine A
ANT: adenine nucleotide transporter
TMRM: tetramethyl rhodamine methyl ester
CM-H_2_DCFDA: chloromethyl 2′,7′-dichlorodihydrofluorescein diacetate.
