# Supplementary material for: Oxidative stress alters mitochondrial bioenergetics and modifies pancreatic cell death independently of cyclophilin D, resulting in an apoptosis-to-necrosis shift
Source: J Biol Chem. 2018 Apr 6;293(21):8032–47. doi: 10.1074/jbc.RA118.003200 (PMC5971444; doi:10.1074/jbc.RA118.003200)
Supplement: Supporting Information [file supp_293_21_8032__index.html]

Oxidative stress alters mitochondrial bioenergetics and modifies pancreatic cell death independently of cyclophilin D, resulting in an apoptosis-to-necrosis shift — Oxidants, mitochondrial bioenergetics, and MPTP — Supporting Information 

# Oxidative stress alters mitochondrial bioenergetics and modifies pancreatic cell death independently of cyclophilin D, resulting in an apoptosis-to-necrosis shift

## Supporting Information

- Supplemental figure 1
- Supplemental figure 2
