# Supplementary material for: Oxidative stress alters mitochondrial bioenergetics and modifies pancreatic cell death independently of cyclophilin D, resulting in an apoptosis-to-necrosis shift
Source: J Biol Chem. 2018 Apr 6;293(21):8032–47. doi: 10.1074/jbc.RA118.003200 (PMC5971444; doi:10.1074/jbc.RA118.003200)
Supplement: Supporting Information [file supp_RA118.003200_figure_S1.pdf]

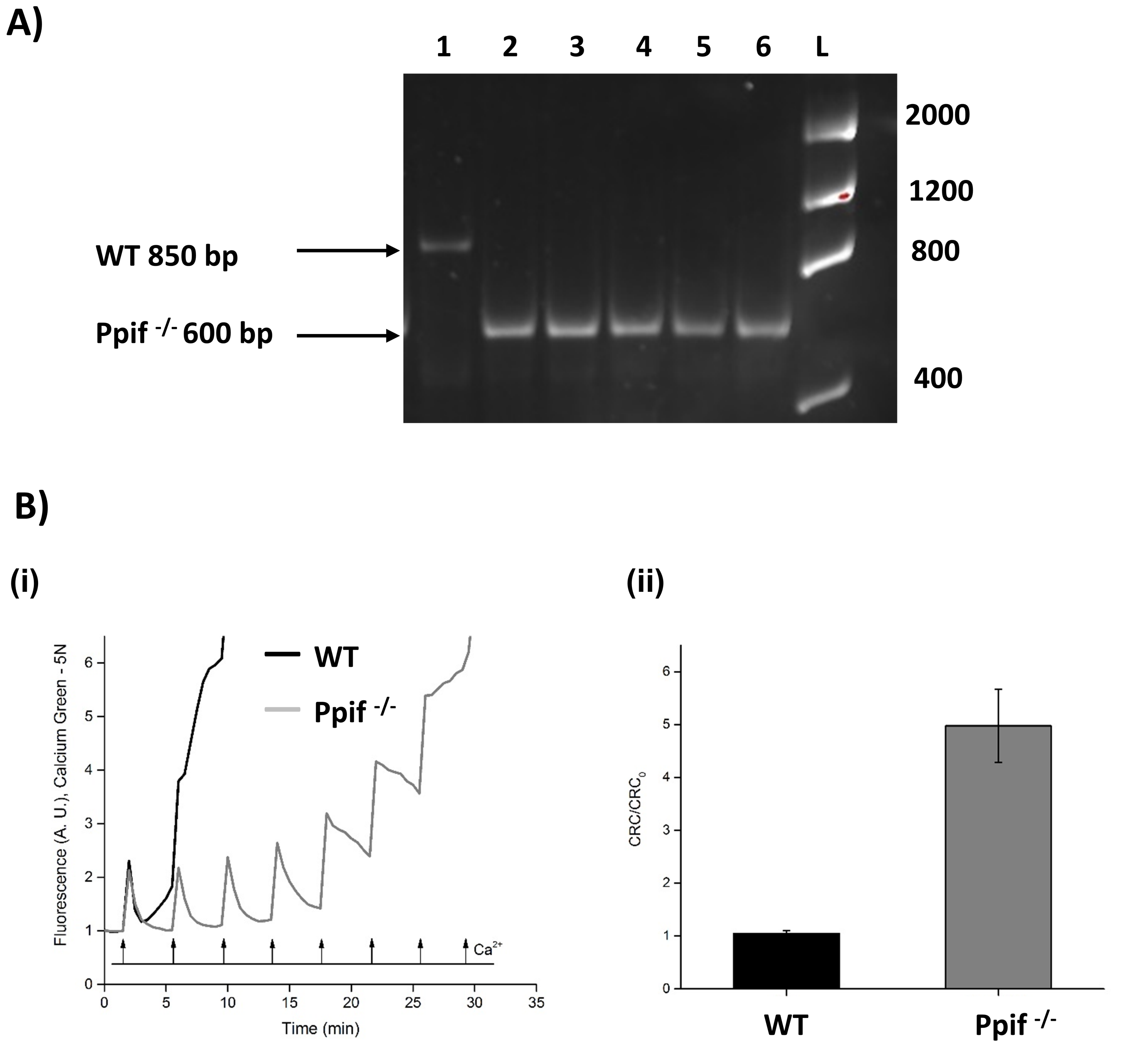

Figure S1. Cyclophilin D deletion (*Ppif* $^{-/-}$) prevents MPTP formation in a calcium retention capacity (CRC) assay. A) Gel electrophoresis analysis of PCR amplification of WT and *ppif* $^{-/-}$ mice. The wild-type allele was amplified as a band ~850bp and the null allele ~600bp. Lane 1 shows a WT animal, lanes 2-6 show Ppif$^{-/-}$ mice (last lane shows a molecular ladder). B) (i) Representative traces from the CRC assay in isolated liver mitochondria from WT and *Ppif* $^{-/-}$ mice. Calcium was added every 4 minutes to mitochondria (0.25 mg/ml) in the presence of the fluorescent Ca^{2+} indicator Calcium Green 5N ($\lambda_{ex}/\lambda_{em}$: 490/530 nm). Repeated Ca^{2+} challenges induced fluorescent spikes that increased until collapse of the MPTP, indicated by permanent high fluorescence. The time to maximum fluorescence correlates with inhibition of the MPTP. B) (ii) Bar graph of the CRC/CRC_0 of WT and *Ppif* $^{-/-}$ mitochondria, where CRC_0 is the number of peaks obtained with the WT mitochondria ($n \geq 3$).
