# Supplementary material for: Oxidative stress alters mitochondrial bioenergetics and modifies pancreatic cell death independently of cyclophilin D, resulting in an apoptosis-to-necrosis shift
Source: J Biol Chem. 2018 Apr 6;293(21):8032–47. doi: 10.1074/jbc.RA118.003200 (PMC5971444; doi:10.1074/jbc.RA118.003200)
Supplement: Supporting Information [file supp_RA118.003200_Figure_S2.pdf]

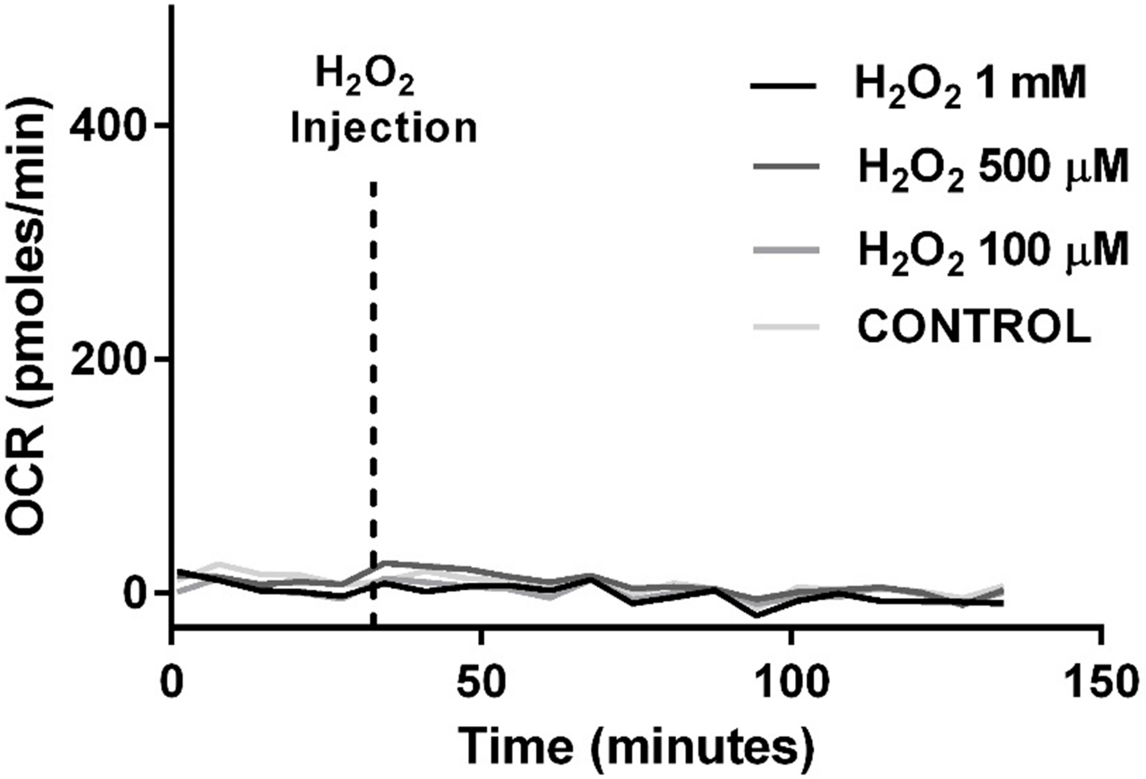

Figure S2. Effects of H_2O_2 on basal OCR in a Seahorse Flux assay in the absence of cells. Control experiments in which H_2O_2 was applied to empty wells in the Seahorse XF24. The results show that basal fluorescence (OCR sensor) was unaltered by the presence of the oxidant (n = 3).
